# The Effect of Dysregulation of tRNA Genes and Translation Efficiency Mutations in Cancer and Neurodegeneration

**DOI:** 10.3389/fgene.2012.00201

**Published:** 2012-10-12

**Authors:** Tamir Tuller

**Affiliations:** ^1^Department of Biomedical Engineering, Tel Aviv UniversityRamat Aviv, Israel

## tRNA Levels and Codon Bias Affect the Fidelity and Efficiency of Translation

The two major steps of gene translation are transcription of the gene to mRNA molecules, and their translation to proteins by the ribosomes. The elongation part of translation includes the iterative decoding of the gene codons by the ribosome with the aid of tRNA molecules. Each codon is recognized by a set of tRNA molecules that are charged with the amino acid encoded by it (Alberts et al., 2002), and the translation time of a codon is usually positively correlated with the abundance of the tRNA molecules recognizing it. Thus, increasing expression levels of the tRNA molecules recognizing a codon, or replacing a codon with a different one recognized by tRNA molecules with higher expression levels, should usually have positive effect on its translation rate (Gustafsson et al., 2004; Tuller et al., 2010).

Missense errors in translation occur at a rate of 1 per 10^3^–10^4^ (Ogle and Ramakrishnan, 2005; Kramer and Farabaugh, 2007), i.e., assuming average protein length of 400 codons, around 18% of the proteins contain at least one missense substitution. In addition, roughly 10–50% of random substitutions disrupt protein function, usually due to loss of folding mutations (Pakula and Sauer, 1989; Markiewicz et al., 1994; Guo et al., 2004; Bloom et al., 2006). It is known that the speed by which codons are translated can also affect the rate of missense translation errors, and thus the folding of the translated protein, resulting in misfolded toxic proteins (Akashi, 1994; Bloom et al., 2006; Zhou et al., 2009). Thus, the probability of missense translation errors of codons that are recognized by tRNA genes with lower concentrations is usually higher than in codons with higher concentrations, since in these cases with higher probability a wrong tRNA replaces the right one (Akashi, 1994; Zhou et al., 2009).

## Global Up-Regulation of tRNA Levels in Cancer

It is known that in many cancerous cells there is an increased growth rate that is regulated by signals related to proliferation, metabolism, and protein synthesis (White, 2005; Gillies et al., 2008; Jones and Thompson, 2009; Mei et al., 2010b; Cairns et al., 2011). This phenomenon may be partially caused by global up-regulation of tRNA molecules. The trigger for these global signals can be the down-regulation of retinoblastoma proteins, p53 and ARF, which cause up-regulation of RNA polymerases I and III, and oncoproteins such as Myc that stimulate the transcription of rRNA and tRNA genes (Cabarcas and Schramm, 2011).

Indeed, the effect of tRNA on tumorigenesis has been previously reported (Berns, 2008; Pavon-Eternod et al., 2009). For example, Pavon-Eternod et al. (2009) used tRNA chips for measuring the expression levels of tRNA molecules to show that in breast cancer there is global over-expression of tRNA species. Specifically, the expression levels of nuclear-encoded tRNAs increase by up to threefold, and mitochondrial-encoded tRNAs increase by up to fivefold in breast cancer. It was also shown that in general these changes maintain the ranking of the expression levels of tRNA genes, as there is significant correlation between the tRNA levels in cancerous and healthy cells (Mahlab et al., 2012). Similar results were obtained for other components of the translation machinery (such as aminoacyl-tRNA synthetases; Vellaichamy et al., 2009).

In this subsection we emphasized the *global* changes in the expression levels of tRNA genes. However, it was also reported that specific pathways and genes relevant to cancer undergo increased differential regulation of translation in cancer due to point mutations in specific genes’ coding sequences, or genomic changes that effect tRNA levels. For example, it was shown that tRNA isoacceptor over-expression may increase the translational efficiency of genes relevant to cancer development, progression (Pavon-Eternod et al., 2009), and apoptosis (Mei et al., 2010a,b). In addition, it was demonstrated that many of the gain-of function and dominant-negative mutations in the tumor suppressor gene TP53 increase its translation efficiency also when considering the cancerous changes in the tRNA pool (Waldman et al., 2009).

## tRNA Genes, Translation Fidelity, and Neurodegeneration

As mentioned above, mistranslation-induced protein misfolding is a dominant constraint on coding sequence evolution (Drummond and Wilke, 2008).

Misfolded proteins is one of the causes of neurodegeneration (Dobson, 2003; Selkoe, 2003; Ross and Poirier, 2004; Lee et al., 2006). For example, it was shown that low levels of mischarged tRNAs can lead to an intracellular accumulation of misfolded proteins in neurons (Lee et al., 2006); similarly, mutations in other components of the translation machinery, such as Aminoacyl-tRNA synthetases, may lead to similar problems (Antonellis and Green, 2008). Indeed, it was shown that mutations in broadly expressed genes involved in translation and protein folding produce brain-specific phenotypes (Zhao et al., 2005; Lee et al., 2006), suggesting that neural tissues are more sensitive to protein misfolding; thus, this sensitivity corresponds to lower mutation rates (or evolutionary rates) in animal neuronal genes (Zhang and Li, 2004; Lee et al., 2006; Wang et al., 2007; Drummond and Wilke, 2008; Tuller et al., 2008). These results suggest that the disruption of translational fidelity in terminally differentiated neurons leads to the accumulation of misfolded proteins and cell death, and provides a novel mechanism underlying neurodegeneration.

## The Diagnostic Potential of tRNA Genes and Coding Sequence Mutations and Corresponding Challenges

The results reviewed in this paper suggest that the expression levels of tRNA genes can be used as biomarkers for diseases such as cancer. One challenge related to this point is to develop robust and efficient approaches for measuring tRNA levels. This is not trivial due to two major reasons: first, tRNA molecules undergo many RNA modifications making the mapping of their deep sequencing reads more challenging (Gustilo et al., 2008; Mahlab et al., 2012). Second, the strong folding of the tRNA molecules decreases their hybridization to DNA chips. Currently, one of the most reliable approaches for measuring tRNA levels is by DNA chips designed specifically for this purpose by Prof. Tao Pan (Dittmar et al., 2006; Pavon-Eternod et al., 2009). However other methods for measuring tRNA levels, e.g., by using liquid chromatography mass spectrometry and signature digestion products (Castleberry and Limbach, 2010), are available.

**Figure 1:**
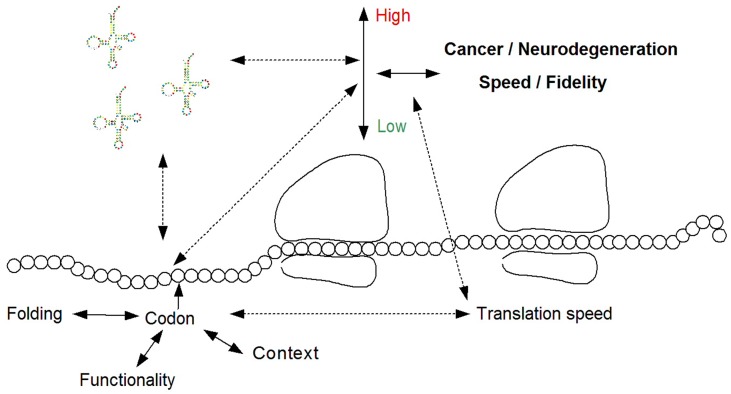
**The relation between translation efficiency and the pathogenesis of cancer and neurodegeneration, and the possible diagnosis of these diseases based on tRNA molecules, and/or the effect of the tRNA pool on mutations and SNPs**. Such a diagnostic tool should consider the tRNA pool and the adaptation of the mutated codon to it, the biophysics of translation including the context of the codon and the effect of the mutation on the mRNA folding, and the functionality of the mutated protein.

In addition, diagnosis related to cancer and neurodegeneration can be performed based on non-synonymous, but also synonymous mutations and *SNPs*, by predicting their effect on the translation rate of a gene. Such a diagnostic tool should consider the adaptation of the mutated codon, or the codon with a SNP, to the human tRNA pool; this can be done by measuring the tRNA levels in the relevant tissue (as mentioned above) or by using a proxy such as the tRNA copy number (Mahlab et al., 2012). In addition, the diagnostic tool should consider amongst others the region of the mutation/SNP within the coding sequence, the codons, and nucleotides surrounding it, and also the effect of the relevant codon on the folding of the mRNA and the charge of the protein it encodes (Tuller et al., 2011). To this end, biophysical predictors of translation efficiency can be employed (Tuller et al., 2010). Finally, the diagnosis should be based on the functionality of the gene; for example, the effect of elevated/decreased adaptation of a codon to the tRNA pool is expected to be opposite for tumor suppressors and oncogenes (Figure 1).
